# Molecular Survey of *Rickettsia raoultii* in Ticks Infesting Livestock from Pakistan with Notes on Pathogen Distribution in Palearctic and Oriental Regions

**DOI:** 10.3390/vetsci10110636

**Published:** 2023-10-29

**Authors:** Shehla Shehla, Mashal M. Almutairi, Abdulaziz Alouffi, Tetsuya Tanaka, Shun-Chung Chang, Chien-Chin Chen, Abid Ali

**Affiliations:** 1 Department of Zoology, Abdul Wali Khan University Mardan, Khyber Pakhtunkhwa Pakistan, Mardan 23200, Pakistan; 2 Department of Pharmacology and Toxicology, College of Pharmacy, King Saud University, Riyadh 11451, Saudi Arabia; 3 King Abdulaziz City for Science and Technology, Riyadh 12354, Saudi Arabia; 4 Laboratory of Infectious Diseases, Joint Faculty of Veterinary Medicine, Kagoshima University, Kagoshima 890-0065, Japan; 5 Department of Emergency Medicine, Ditmanson Medical Foundation Chia-Yi Christian Hospital, Chiayi 60002, Taiwan; 6 Department of Pathology, Ditmanson Medical Foundation Chia-Yi Christian Hospital, Chiayi 60002, Taiwan; 7 Department of Cosmetic Science, Chia Nan University of Pharmacy and Science, Tainan 717, Taiwan; 8 Rong Hsing Research Center for Translational Medicine, National Chung Hsing University, Taichung 402, Taiwan; 9 Department of Biotechnology and Bioindustry Sciences, College of Bioscience and Biotechnology, National Cheng Kung University, Tainan 701, Taiwan

**Keywords:** rickettsial DNA, spotted fever group, *Hyalomma turanicum*, phylogenetic analyses, Pakistan

## Abstract

**Simple Summary:**

Ticks are chelicerate arthropods that feed on blood and infest all vertebrates except fish and transmit different disease-causing agents including *Rickettsia* spp. to domestic and wild animals as well as humans. In the present study, we aimed to molecularly screen and genetically characterize *Rickettsia* spp. in various tick species infesting camels, sheep, and goats from five districts (Kohat, Dera Ismail Khan, Lower Dir, Bajaur, and Mansehra) of Khyber Pakhtunkhwa province, Pakistan. A total of 8/148 (5.4%) ticks, including four *Hyalomma turanicum*, two *Haemaphysalis cornupunctata*, one *Haemaphysalis montgomeryi*, and one *Haemaphysalis bispinosa*, were found positive for *Rickettsia* sp. The phylogenetic analysis of detected *Rickettsia* sp. based on three genetic markers (*gltA, ompA,* and *ompB*) revealed 100% identity with *Rickettsia raoultii*, clustered with its corresponding species reported in China, Russia, USA, Turkey, Denmark, Austria, Italy, and France. Further comprehensive studies on molecular and serosurveillance of various *Rickettsia* spp. in different ticks should be conducted in the region to understand the zoonotic threats due to these pathogens.

**Abstract:**

Ticks are hematophagous ectoparasites that transmit different pathogens such as *Rickettsia* spp. to domestic and wild animals as well as humans. Genetic characterizations of *Rickettsia* spp. from different regions of Pakistan are mostly based on one or two genetic markers and are confined to small sampling areas and limited host ranges. Therefore, this study aimed to molecularly screen and genetically characterize *Rickettsia* spp. in various tick species infesting camels, sheep, and goats. All the collected tick specimens were morphologically identified, and randomly selected tick species (148) were screened molecularly for the detection of *Rickettsia* spp. by amplifying three rickettsial DNA fragments, namely, the citrate-synthase gene (*gltA*), outer-membrane protein A (*ompA*), and outer-membrane protein B (*ompB*). After examining 261 hosts, 161 (61.7%) hosts were found infested by 564 ticks, including 287 (50.9%) nymphs, 171 (30.3%) females, and 106 (18.8%) males in five districts (Kohat, Dera Ismail Khan, Lower Dir, Bajaur, and Mansehra). The highest occurrence was noted for *Hyalomma dromedarii* (number = 72, 12.8%), followed by *Haemaphysalis sulcata* (n = 70, 12.4%), *Rhipicephalus turanicus* (n = 64, 11.3%), *Rhipicephalus microplus* (n = 55, 9.7%), *Haemaphysalis cornupunctata* (n = 49, 8.7%), *Hyalomma turanicum* (n = 48, 8.5%), *Hyalomma isaaci* (n = 45, 8.0%), *Haemaphysalis montgomeryi* (n = 44, 7.8%), *Hyalomma anatolicum* (n = 42, 7.5%), *Haemaphysalis bispinosa* (n = 38, 6.7%), and *Rhipicephalus haemaphysaloides* (n = 37, 6.6%). A subset of 148 ticks were tested, in which eight (5.4%) ticks, including four *Hy. turanicum*, two *Ha. cornupunctata*, one *Ha. montgomeryi*, and one *Ha. bispinosa*, were found positive for *Rickettsia* sp. The *gltA*, *ompA*, and *ompB* sequences revealed 100% identity and were phylogenetically clustered with *Rickettsia raoultii* reported in China, Russia, USA, Turkey, Denmark, Austria, Italy, and France. Additionally, various reports on *R. raoultii* from Palearctic and Oriental regions were summarized in this study. To the best of our knowledge, this is the first report regarding genetic characterization and phylogenetic analysis of *R. raoultii* from Pakistan. Further studies to investigate the association between *Rickettsia* spp. and ticks should be encouraged to apprise effective management of zoonotic consequences.

## 1. Introduction

Ticks carry and transmit a wide range of pathogens comprising viruses, fungi, protozoans, and bacteria [1,2,3]. The genus *Rickettsia* is comprised of obligate Gram-negative bacteria, is distributed worldwide, and can cause rickettsiosis in hosts including domestic and wild animals, as well as humans [2,4]. Arthropod vectors such as ticks, fleas, mites, and lice may transmit *Rickettsia* spp.; however, the competent vectors for its propagation are mostly Ixodid ticks [5]. Tick-borne rickettsiosis is a known vector-borne zoonotic disease [2], and the majority of the tick-borne *Rickettsia*e belong to the spotted fever group (SFG) of *Rickettsia* [6]. So far, almost 33 different *Rickettsia* spp. and 19 different *Candidatus* (*Ca*) Rickettsia spp. in the SFG group have been identified globally [7,8,9,10,11].

*Rickettsia raoultii* was first detected in *Rhipicephalus pumilio* and *Dermacentor nuttalli* ticks from the former Soviet Union in 1999 [12]. Then, it was isolated from *Dermacentor silvarum* ticks in 2008 [13]. It has been detected in various tick species including *Dermacentor marginatus*, *Dermacentor nuttalli*, *Dermacentor reticulatus*, *Dermacentor silvarum*, *Haemaphysalis longicornis*, *Haemaphysalis erinacei*, *Haemaphysalis concinna*, *Ixodes persulcatus*, *Ixodes canisuga*, *Ixodes ricinus*, and *Rhipicephalus sanguineus* [14,15,16,17,18,19,20,21,22]. Later, *R. raoultii* was also isolated from embryo-derived tick cell lines originating from *Rhipicephalus microplus* [23] and *Rhipicephalus sanguineus* [24]. Additionally, various fleas including *Ctenocephalides felis* collected from goats have been identified as potential carriers of *R. raoultii* [25]. This *Rickettsia* sp. has been detected in the blood and various tissues of animals including heart, liver, spleen, lung, and kidney [19,26,27,28,29,30].

In humans, *R. raoultii* causes SENLAT (scalp eschars and neck lymphadenopathy after a tick bite) syndrome, initially named DEBONEL (*Dermacentor*-borne necrotic erythema and lymphadenopathy) or TIBOLA (tick-borne lymphadenopathy after a tick bite) [22,31,32]. Meningeal syndrome and neurological abnormalities such as eyelid droop and elevated cerebrospinal pressure have also been documented as clinical symptoms of *R. raoultii* infections [32,33]. Although generally linked with mild infections, more severe infections with leukopenia, thrombocytopenia, and septic features have also been described, indicating different degrees of virulence or susceptibility to *R. raoultii* [34]. The pathogenicity of *R. raoultii* has been reported in Spain, France, Slovakia, Poland, and China [29,32,33,34,35,36]. Globally, *R. raoultii* has been detected serologically and molecularly in ticks, fleas, animals, humans, and vegetation.

Pakistan is an agricultural country, and livestock play an important role in its economy, as different animals are a major source of income for rural inhabitants. However, in Pakistan, different ticks including *Hyalomma* spp., *Rhipicephalus* spp., *Amblyomma* spp., *Ixodes* spp., *Ornithodoros* spp., *Nosomma* spp., and *Haemaphysalis* spp. have been reported infesting livestock and wild animals, and these ticks can transmit different pathogens such as Anaplasma spp., *Rickettsia* spp., *Babesia* spp., *Theileria* spp., and *Coxiella* spp. [37,38,39,40,41,42,43,44,45]. In Pakistan, some *Rickettsia* spp. have been reported in ticks infesting equids, bovines, and wild animals [37,39,46,47], and these *Rickettsia* species were reported based on targeting only one or two rickettsial markers. Hence, there is a dearth of information regarding the genetic characterization of *Rickettsia* spp. in different tick species infesting camels, sheep, and goats. Novel *Rickettsia* species of undetermined pathogenicity are continuously detected in ticks, necessitating effective tools to infer their phylogenetic relationships. The present study aims to molecularly characterize the *Rickettsia* spp. in hard ticks infesting camels, sheep, and goats by using three genetic markers in Khyber Pakhtunkhwa (KP), Pakistan.

## 2. Materials and Methods

### 2.1. Ethical Approval

This study was approved by the members of graduate study committee and Advance Studies Research Board (AWKUM/CE/SC/2022/12041) of the Zoology Department, Abdul Wali Khan University Mardan, Pakistan. Verbal and written permission was obtained from livestock owners before examining their animals for the collection of ticks.

### 2.2. Study Area

**Figure 1:**
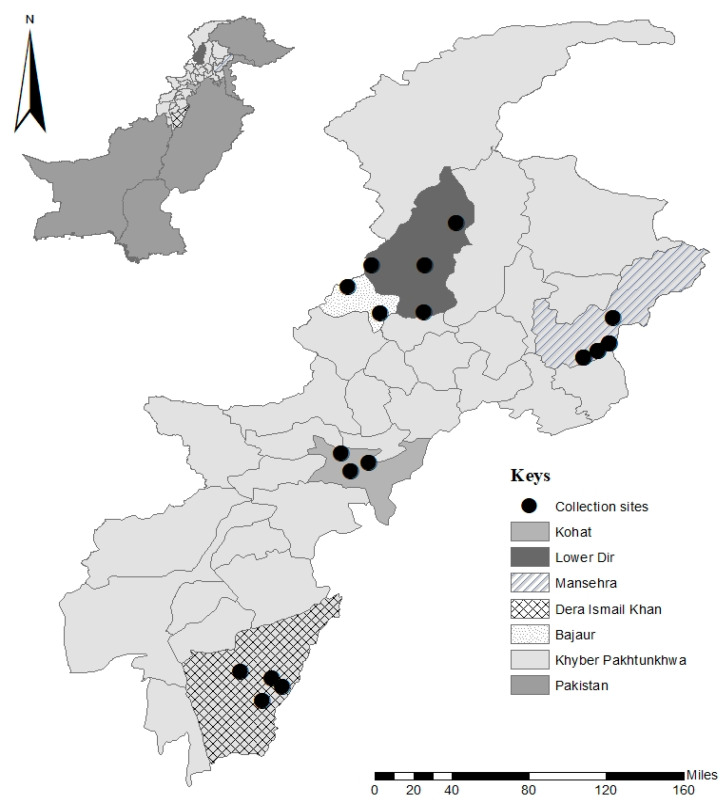
Map showing collection sites in the study area where ticks infesting camels, sheep, and goats were collected for the detection of *Rickettsia* spp.

The current study was performed in five districts of the KP province, including Kohat (33°33′36.0″ N 71°28′31.5″ E), Dera Ismail Khan (D.I Khan) (31°51′02.9″ N 70°53′28.5″ E), Lower Dir (34°54′10.6″ N 71°47′21.6″ E), Bajaur (34°48′11.0″ N 71°31′08.3″ E), and Mansehra (34°19′40.4″ N 73°11′56.1″ E). Different hosts including camels, sheep, and goats were examined for the collection of ticks from July 2020–June 2021. The latitudes and longitudes of the tick collection sites were collected via the Global Positioning System (GPS) and imported to Microsoft Excel V. 2013 for processing. The study area map (Figure 1) was designed in ArcGIS V. 10.3.1 (ESRI, Redlands, CA, USA).

### 2.3. Ticks Collection and Identification

Tick collection was performed on camels, sheep, and goats in the selected study area. Ticks were collected from the aforementioned hosts by examining the entire body, and they were found regardless of the specific locations and times within the targeted survey districts in various farms, open fields, and freely moving animals in pastures. Collection was performed only once for each host when tick infestation was detected. Host-based collected ticks were separately stored in micro-tubes labelled with the collection sites and specified host. Tick specimens were washed with distilled water and preserved in 100% ethanol for further processing.

Collected tick specimens were identified morphologically using a stereomicroscope (SZ61, Olympus, Japan) by following the available standard keys [39,48,49,50,51,52]; then, identified ticks were preserved in 100% ethanol until molecular analysis.

### 2.4. Molecular Screening of Rickettsia spp.

A total of 148 (74N, 74F) tick specimens were subjected to DNA extraction for molecular analyses. Individual ticks were crushed and their genomic DNA was extracted through a standard method of phenol-chloroform [53]. Genomic DNA was not extracted from all the morphologically identified ticks because we selected representative ticks of each tick species from their respective host in each district. The extracted DNA was quantified using Nanodrop (Nano-Q, Optizen, Daejeon, Republic of Korea) and stored at −20 °C for further molecular experimentation. The extracted DNA of each individual tick was tested for the presence of *Rickettsia* spp. through a conventional PCR targeting the amplification of fragments of three genes including citrate-synthase (*gltA*), outer-membrane protein A (*ompA*), and outer-membrane protein B (*ompB*). The PCR reaction was performed in 25 µL, having 1 µL of each primer (forward and reverse) (10 µM), 2 µL of genomic DNA (50–100 ng), 12.5 µL of Dream*Taq* MasterMix (2×) (Thermo Fisher Scientific, Inc., Waltham, MA, USA), and 8.5 µL of PCR water (nuclease free). The primers and PCR conditions used for the amplification of the aforementioned fragments are provided in Table 1. After PCR, the amplified products were run on 1.5% agarose gel and the results were visualized under UV light through the Gel Doc system (UVP BioDoc-It imaging system; Analytik Jena AG, Jena, Germany).

### 2.5. Sequences and Phylogenetic Analyses

The manufacturer’s protocol was adopted for the purification of all the amplified PCR products using the GeneClean II Kit (Qbiogene, Illkirch, France), and products were submitted for bidirectional sequencing (Macrogen Inc., Seoul, Republic of Korea) through the Sanger-based method. All the obtained bidirectional sequences were trimmed in SeqMan v. 5.0 (DNASTAR) by removing poor sequencing reads and primer contaminations. The consensus sequences for each fragment (*gltA, ompA,* and *ompB*) were obtained from all the identical trimmed sequences, which were separately subjected to the Basic Local Alignment Search Tool (BLAST) at the National Center for Biotechnology Information (NCBI). Sequences with high identity were downloaded and aligned in BioEdit alignment editor v 7.0.5 along with selected outgroups [57]. Separately, the phylogenetic trees of *gltA*, *ompA*, and *ompB* were constructed in Molecular Evolutionary Genetic Analysis software [58] by following the neighbor-joining method and Tamura–Nei model [59], in which 1000 bootstrap replicates were used for tree reliability [58].

### 2.6. Literature Search and Selection Criteria

We performed a literature search using databases including Science Direct, Web of Science, Google Scholar, and PubMed. Various keywords including tick(s), small and large ruminant(s), livestock, livestock-holder(s), farmer(s), worker(s), human(s), *R. raoultii*, Ca. *R. raoultii*, *R*. *conorii* subsp. *raoultii*, molecular characterization, phylogenetic analysis, and the specific country names were used in the aforementioned databases. Combinations of keywords were used to download research publications, review articles, short communications, and case-reports regarding *R. raoultii*. A minimum of one report of *R. raoultii* from each country, as well as all the previously reported human cases of *R. raoultii* from Palearctic and Oriental regions, were included in the current study. All this literature-based data was retrieved in July 2023 (Table 2).

## 3. Results

### 3.1. Ticks and Hosts

Overall, 261 hosts, including camels (n = 99/261, 37.9%), sheep (n = 85/261, 32.6%), and goats (n = 77/261, 29.5%), were inspected in the aforementioned five selected districts for tick collection, among which 161/261 (61.7%) hosts were found infested with ticks, among which camels were highly infested (n = 64/99, 64.6%), followed by goats (n = 49/77, 63.6%) and sheep (48/85, 56.5%). The infestation rate of various hosts was highest in Kohat (number = 40/55, 72.7%), followed by D.I. Khan (n = 30/48, 62.5%), Mansehra (n = 35/59, 59.3%), Bajaur (n = 28/50, 56.0%), and Lower Dir (n = 28/49, 57.1%). A total of 564 ticks were collected from the aforementioned hosts, and 11 different tick species belonging to three genera (*Haemaphysalis*, *Hyalomma*, and *Rhipicephalus*) were morphologically identified. In the current study, the highest number of ticks was collected from sheep (n = 240/564, 42.5%), followed by goats (n = 186/564, 33.0%) and the lowest number of ticks was collected from camels (n = 138/564, 24.5%). *Hyalomma dromedarii* ticks were recorded as having the highest occurrence (n = 72/564, 12.8%), followed by *Haemaphysalis sulcata* (n = 70/564, 12.4%), *Rhipicephalus turanicus* (n = 64/564, 11.3%), *Rh. microplus* (n = 55/564, 9.7%), *Ha. cornupunctata* (n = 49/564, 8.7%), *Hyalomma turanicum* (n = 48/564, 8.5%), *Hyalomma isaaci* (n = 45/564, 8.0%), *Haemaphysalis montgomeryi* (n = 44/564, 7.8%), *Hyalomma anatolicum* (n = 42/564, 7.5%), *Haemaphysalis bispinosa* (n = 38/564, 6.7%), and *Rhipicephalus haemaphysaloides* (n = 37/564, 6.6%) (Table 3).

### 3.2. Molecular Screening of Rickettsia spp.

DNA extracted from 148 (74N, 74F) identified ticks was tested for *Rickettsia* spp. Ticks (n = 8/148, 5.4%) were found positive for *Rickettsia* sp. in Kohat (n = 2/36, 5.6%), D.I Khan (n = 2/32, 6.3%), Lower Dir (n = 2/24, 8.3%), Bajaur (n = 1/28, 3.6%), and Mansehra (n = 1/28, 3.6%). Four tick species were found positive for rickettsial DNA, including *Hy. turanicum*, *Ha. cornupunctata*, *Ha. montgomeryi* and *Ha. bispinosa*. *Rickettsia* sp. was detected in *Hy. turanicum* infesting camels and sheep in the Kohat and D.I Khan districts, respectively. Additionally, *Rickettsia* sp. was also detected in *Ha. cornupunctata*, *Ha. sulcata*, and *Ha. montgomeryi* infesting sheep in district Lower Dir, Bajaur, and Mansehra, respectively. However, no rickettsial DNA was detected in *Hy. dromedarii*, *Hy. isaaci*, *Hy. anatolicum*, *Rh. turanicus*, *Rh. haemaphysaloides*, *Rh. microplus*, and *Ha. sulcata* (Table 3).

### 3.3. Sequence and Phylogenetic Analysis

**Figure 2:**
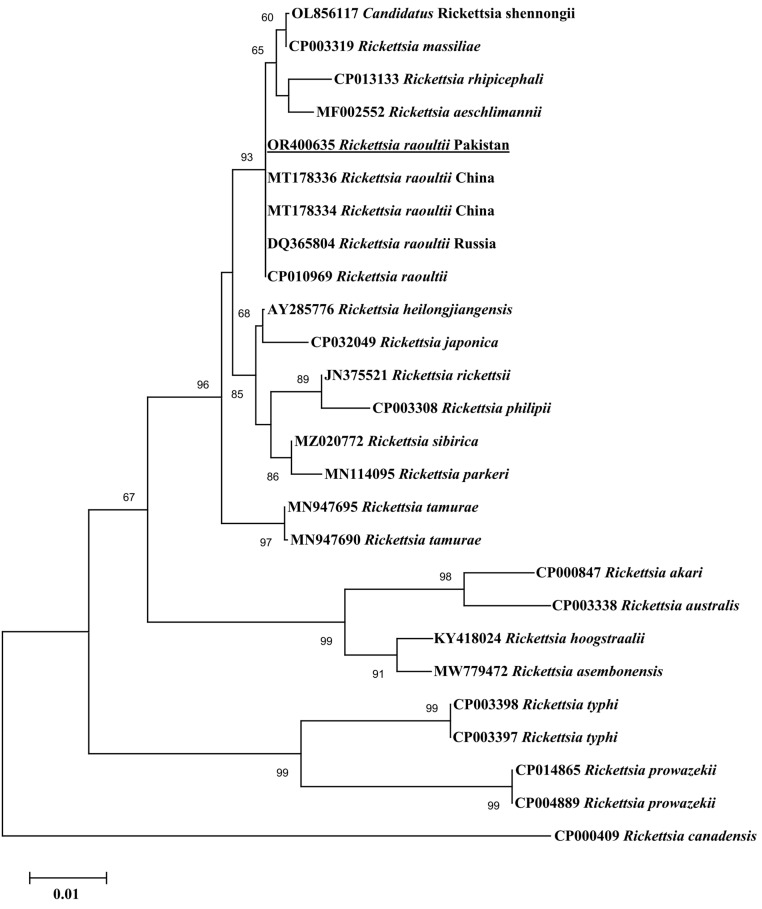
Phylogeny was inferred based on rickettsial gltA fragments using the neighbor-joining method. GenBank accession numbers are followed by the species and country names at each terminal taxon. Rickettsia canadensis (CP000409) was taken as an outgroup using 1000 bootstrap values at each node. The present gltA sequence (accession no. OR400635) for R. raoultii is marked with bold and underlined font.

**Figure 3:**
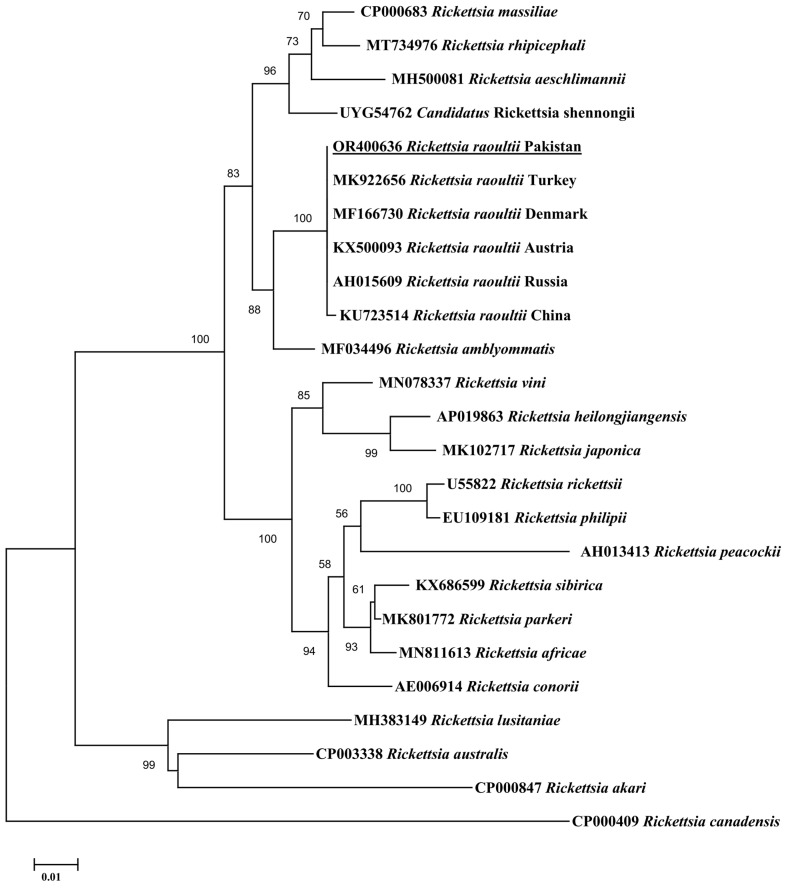
Phylogeny was inferred based on rickettsial ompA fragments using the neighbor-joining method. GenBank accession numbers are followed by the species and country names at each terminal taxon. Rickettsia canadensis (CP000409) was taken as an outgroup using 1000 bootstrap values at each node. The present ompA sequence (accession no. OR400636) for R. raoultii is marked with bold and underlined font.

**Figure 4:**
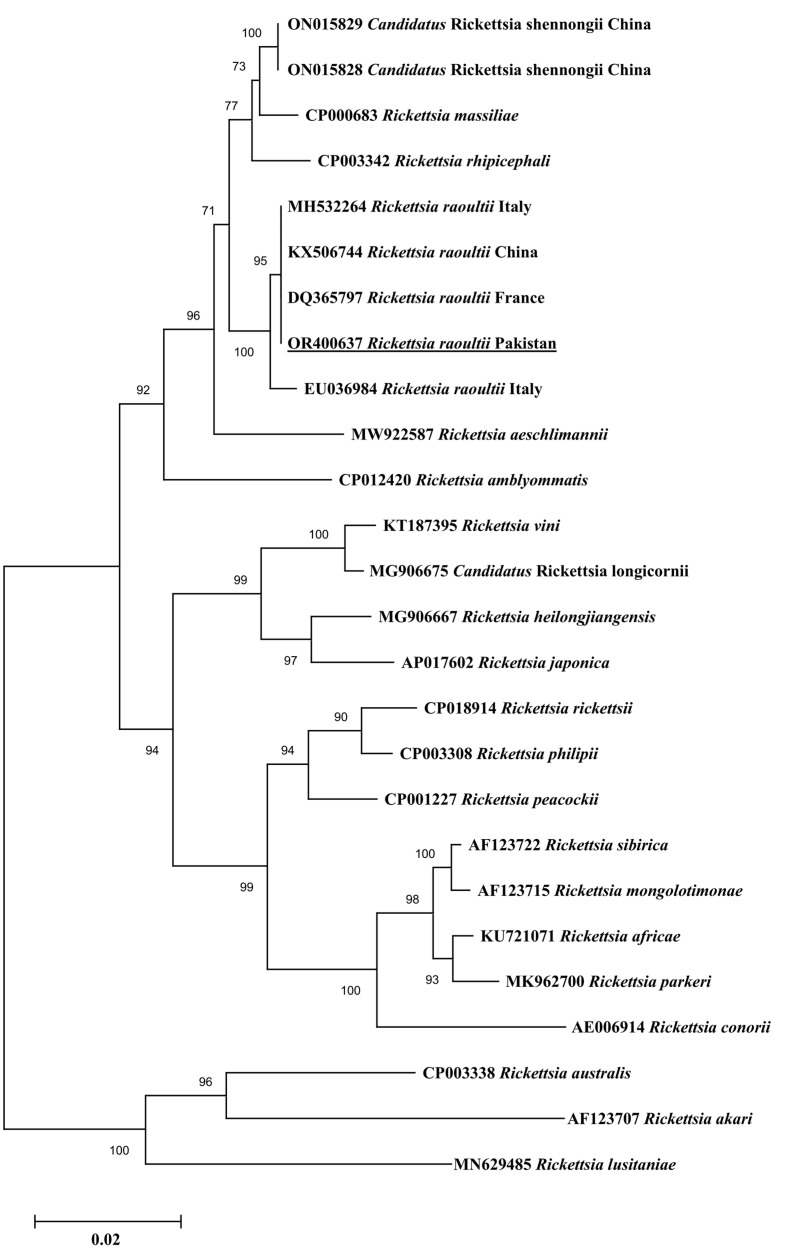
Phylogeny was inferred based on rickettsial ompB fragments using the neighbor-joining method. GenBank accession numbers are followed by species and country names at each terminal taxon. A group of sequences of Rickettsia lusitaniae (MN629485), Rickettsia australis (CP003338), and Rickettsia akari (AF123707) were taken as outgroups using 1000 bootstrap values at each node. The present gltA sequence of (accession no. OR400637) R. raoultii is marked with bold and underlined font.

In the BLAST analysis, the consensus sequence of *gltA* obtained for *Rickettsia* sp. revealed 100% identity with *R. raoultii* reported in China (MT178334─MT178338), Russia (DQ365804), and the USA (CP010969). The consensus sequence of *ompA* showed 100% identity with *R. raoultii* reported in Turkey (MK922656), Denmark (MF166730), Austria (KX500093), China (KX723514), and Russia (AH015609). The consensus sequence of *ompB* showed 100% identity with *R. raoultii* reported in Italy (MH532264), China (KX506744), and France (DQ365797). In the phylogenetic tree, the *gltA* sequence of *R. raoultii* clustered with corresponding species reported in China, Russia, and the USA (Figure 2). The *ompA* sequence of *R. raoultii* clustered with the corresponding species reported in Turkey, Denmark, Austria, Russia, and China (Figure 3). The *ompB* sequence of *R. raoultii* clustered with the corresponding species reported in Russia, Italy, and China (Figure 4). The obtained sequences of *R. raoultii* were submitted to GenBank under the accession numbers OR400635 (*gltA*), OR400636 (*ompA*), and OR400637 (*ompB*).

## 4. Discussion

Ticks pose health threats to humans and animals, as they can transmit numerous pathogens including SFG *Rickettsia* spp. [1,2,3]. Previously, potential health risks to humans posed by rickettsial agents have been worsened by some *Rickettsia* spp. including *R. raoultii*, which can cause rickettsiosis in humans in different regions of the world [22,32,74,91]. Camels, sheep, and goats are considered as human companions, and these animals have been identified as a notable reservoir hosts for many *Rickettsia* spp., which might play an important role in the natural transmission cycle and dispersal of different *Rickettsia* spp. [2,92,112]. Hence, a regular surveillance of various rickettsial agents carried by ticks infesting the aforementioned hosts is essential to minimize public health risks. Herein, *R. raoultii* was molecularly analyzed via standard genetic markers in eleven morphologically identified tick species infesting camels, sheep, and goats in five districts of KP, Pakistan, and *R. raoultii* was identified in four tick species including *Hy. turanicum*, *Ha. cornupunctata*, *Ha. montgomeryi*, and *Ha. bispinosa*.

Host density in the herds ultimately increases the chances of tick infestation compared to those animals kept alone due to the possibility of infestation by detached host-questing ticks in the herd area [46]. In current study, ticks of different genera including *Haemaphysalis, Rhipicephalus,* and *Hyalomma* were found infesting the aforementioned hosts, which were sharing their habitats, thus enhancing the possibilities for an available wide host range. Here, *Hy. dromedarii* (n = 72/564, 12.8%) was the most prevalent tick compared to other tick species because the highest number of camels (n = 99/261, 37.9%) was examined compared to other hosts. Previous reports regarding the tick abundance on camels have shown that *Hy. dromedarii* is the most prevalent tick species of dromedary camels because this tick is not influenced by any season, and hence shows a preponderance on camels during both dry and wet seasonal conditions [113,114]. Previous studies have shown that *Hyalomma* ticks can survive successfully in harsh desert regions [115,116]; therefore, the *Hy. dromedarii* ticks were most prevalent because the larger proportion of the study area was composed of desertic plains, arid plains, and arid hilly areas that are suitable for the survival of *Hy*. *dromedarii* ticks. Additionally, these ticks may act as vectors for the transmission of infectious agents to livestock owners [117,118]. Some *Hyalomma*, *Rhipicephalus*, and *Haemaphysalis* tick species infesting humans have been recorded from this region in Pakistan [119].

In Pakistan, different *Rickettsia* spp. have been detected in various tick species including *Ix. kashmiricus*, *Ornithodoros* sp., *Rh. turanicus*, *Rh. haemaphysaloides*, *Rh. microplus*, *Hy. dromedarii*, and *Hy. anatolicum* [37,39,41,46,47]. *Rickettsia raoultii* was detected in four tick species including *Hy. turanicum*, *Ha. sulcata*, *Ha. cornupunctata*, and *Ha. montgomeryi*, and it was previously reported in various tick genera including *Hyalomma*, *Rhipicephalus*, *Dermacentor*, and *Ixodes* in various regions of the world [14,17,81,95]. *Rickettsia raoultii* was detected in different tick species collected from camels, sheep, and goats in the current study. Similarly, *R. raoultii* has been detected in various tick species collected from the aforementioned hosts in different countries including Slovakia, Malaysia, China, Greece, Mongolia, India, Iran, and Turkey [14,25,81,89,92,99,100,108,110]. Our findings provide the first molecular evidence regarding the genetic characterization of *R. raoultii* in *Hy. turanicum* infesting camels, which suggests the possible role of this tick in the dispersal of *R. raoultii* in the specified region. Since adult female and nymph ticks were found positive for *R. raoultii*, there is a possibility that the detected *R. raoultii* was ingested through the blood from infected camels, as this pathogen has been previously detected in the blood of dogs in Germany [77] and Iran [30]. Hence, there is a need to conduct comprehensive serosurveillance and molecular studies on different rickettsial agents in different animals to know the factors responsible for the transmission of these bacteria in the region.

Molecular characterization of *Rickettsia* spp. through the *ompB* gene relies on its outer-membrane locality and the presence of protein epitopes that are common to both typhus and SFG *Rickettsia*e [7,55]. Additionally, it has been previously stated that three rickettsial genes including *gltA*, *ompA*, and *ompB* may be used and are known for the detection of rickettsial agents specifically to investigate the presence of SFG *Rickettsia*e [13,21] and to provide a significant phylogenetic relationship in the *Rickettsia*e [120]. Hence, *R. raoultii* has been previously globally detected in different ticks including *De. marginatus, De. nuttalli, De. silvarum, De. reticulatus, Am. testudinarium, Am. helvolum, Ha. bispinosa, Rh. microplus*, and *Rh. sanguineus* by using three genetic markers: *gltA*, *ompA*, and *ompB* [21,29,79,81,102,121]. The obtained *gltA*, *ompA*, and *ompB* sequences of *R. raoultii* in this study revealed a close evolutionary relationship and were hence phylogenetically clustered with their corresponding species reported in China, Russia, USA, Italy, Turkey, Denmark, and Austria.

Camels, sheep, and goats are considered as human companions and share their household environment, resulting in close contact with each other. *R. raoultii* was identified in different tick species collected from the aforementioned animals; thus, these ticks and their specified hosts may play a role as a source of human infection. Therefore, further serological and molecular studies in the region should be encouraged to understand the zoonotic threats due to these infectious agents.

## 5. Conclusions

*Rickettsia raoultii* has been previously reported in different ticks infesting camels, sheep, and goats globally. Hence, this study genetically characterized *R. raoultii* in four tick species including *Ha. bispinosa*, *Ha. cornupunctata*, *Ha. montgomeryi*, and *Hy. turanicum* infesting camels, sheep, and goats in Pakistan. Additionally, this is the first report regarding the detection of *R. raoultii* in *Hy. turanicum* ticks collected from camels, which suggests that camels may serve as reservoir hosts for *R. raoultii* in the region. Due to close contact between livestock holders and camels, sheep, and goats in the region, there are possibilities for transmission of these bacteria to humans. Thus, surveillance strategies should be adopted to properly investigate these bacteria to minimize any health threats. Further comprehensive studies on molecular and serosurveillance of *Rickettsia* spp. in different ticks should be conducted in the region to understand the zoonotic threats due to these pathogens.

## Figures and Tables

**Table 1:** List of primers used for the amplification of rickettsial fragments in various ticks collected from camels, goats, and sheep in the present study.

Gene	Primer	Sequence	Amplicon Size	PCR Condition	Study
***gltA***	CS-78	GCAAGTATCGGTGAGGATGTAAT	401 bp	95 °C 3 min, 40× (95 °C 15 s, 48 °C 30 s, 72°C 30 s), 72 °C 7 min	[54]
	CS-323	GCTTCCTTAAAATTCAATAAATCAGGAT			
***ompA***	Rrl9O.70	ATGGCGAATATTTCTCCAAAA	532 bp	95 °C 3 min, 35× (95 °C 20 s, 48 °C 30 s, 63 °C 2 min), 72 °C 7 min	[55]
	Rrl9O.602	AGTGCAGCATTCGCTCCCCCT			
***ompB***	120-M59	CCGCAGGGTTGGTAACTGC	862 bp	95 °C 3 min, 40× (95 °C 30 s, 50 °C 30 s, 68 °C 90 s), 68 °C 7 min	[56]
	120-807	CCTTTTAGATTACCGCCTAA			

**Table 2:** A summary of some previously published reports on *Rickettsia raoultii* in ticks, fleas, humans, vegetation, and animals in Palearctic and Oriental regions.

Country/Year of Study	*Rickettsia raoultii*	Tick Species/Source	Hosts/Sources	Identification Method (Serologically/Molecularly)	Genetic Marker (s)	Reference
Morocco/2002–2006	*R. raoultii*	*Dermacentor marginatus*	Livestock, dogs, and vegetation	Molecularly	*gltA, ompA*	[60]
France/2002–2007	*R. raoultii*	Blood	Humans	Serologically and molecularly (sequencing)	*ompA*	[35]
		*Dermacentor* spp.				
Spain/2003–2008	*R. raoultii*	Body fluids and biopsies	Humans	Molecularly (sequencing)	*gltA* and *ompB*	[61]
Japan/2004–2009	*R. raoultii*	*Amblyomma sparsum*	Snakes, tortoises, lizards, and frogs imported from Zambia	Molecularly and phylogenetically	*gltA*	[62]
Slovakia/2004–2010	*R. raoultii*	*De. marginatus*	Vegetation, horses, sheep,	Molecularly and phylogenetically	*gltA, ompA* and *sca4*	[14]
		*Dermacentor reticulatus*	Goats, and dogs			
Turkey/2006	*R. raoultii*	*Hyalomma marginatum*	Humans	Molecularly (sequencing)	*ompA*	[63]
		*De. marginatus*				
Portugal/2006–2009	*R. raoultii*	*De. marginatus*	Vegetation	Molecularly	*gltA, ompA*	[64]
Taiwan/2006–2010	*R. raoultii–*like	liver, spleen, and kidney	*Bandicota indica*	Molecularly (sequencing)	*ompB* and *gltA*	[27]
			*Mus musculus*			
Hungary/2006–2010	*R. raoultii*	*De. marginatus*	Humans	Molecularly (sequencing)	*gltA, ompA,* and*16S rRNA*	[65]
		*De. reticulatus*				
Spain	*R. raoultii*	*De. marginatus*	Humans	Molecularly	*gltA, ompA*	[66]
		Blood				
Georgia/2008–2009	*R. raoultii*	*De. marginatus*	Livestock, rodents	Molecularly	*ompB*	[67]
Malaysia/2008–2011	*R. raoultii*–like	kidney, liver, spleen and heart	Wild rats	Molecularly and phylogenetically	*gltA*	[26]
Belarus/2009	*R. raoultii*	*Ixodes ricinus*	Cows and vegetation	Molecularly and phylogenetically	*ompA*	[68]
		*De. reticulatus*				
Thailand/2009	*R. raoultii–*like	*Amblyomma helvolum*	Lizard	Molecularly and phylogenetically	16S rRNA, *gltA*, and *ompA*	[69]
Korea/2010–2015	*R. raoultii*	*Haemaphysalis longicornis*	Dogs	Molecularly and phylogenetically	16S rRNA	[70]
Germany/2010–2011	*R. raoultii*	*De. reticulatus*	*Myodes glareolus*	Molecularly	*gltA*	[71]
		*Ix. ricinus*				
		Fleas				
Czech Republic/2010–2011	*R. raoultii*	*De. reticulatus*	Vegetation	Molecularly	*gltA*	[72]
Romania/2011–2012	*R. raoultii*	Blood	Humans	Serologically	*–*	[73]
Slovakia/2011–2020	*R. raoultii*	Blood	Humans	Serologically	*–*	[74]
				Molecularly	*gltA, 23S rRNA,* and *ompB*	
Hungary/2011–2012	*R. raoultii*	*De. reticulatus*	Vegetation	Molecularly	*gltA*	[75]
China/2012	*R. raoultii*	*Dermacentor silvarum*	Humans	Molecularly and phylogenetically	*gltA* and *ompA*	[76]
		Blood				
Germany	*R. raoultii*	Blood	Dogs	Serologically		[77]
Mongolia	*R. raoultii*	*Dermacentor nuttalli*	Vegetation	Molecularly and phylogenetically	*ompB*, *gltA*	[78]
Laos/2012–2014,	*R. raoultii*	*Amblyomma testudinarium*		Molecularly and phylogenetically	*ompA*, *gltA*, *ompB*, and *17–kDa*	[79]
		*Haemaphysalis*				
Spain/2012–2019	*R. raoultii*	*De. reticulatus*	Cantabrian brown bear	Molecularly and phylogenetically	*gltA, ompA*	[80]
Malaysia/2012–2013	*R. raoultii*–like	*Haemaphysalis bispinosa*	Cattle, sheep	Molecularly and phylogenetically	*gltA*, *ompA* and *ompB*	[81]
		*Haemaphysalis* spp.	Chicken, Dogs			
		*Rhipicephalus microplus*	Cattle			
		*Rhipicephalus sanguineus*	Dogs			
Malaysia/2013	*R. raoultii*–like	Blood	Human	Molecularly	*gltA*, *ompB*	[28]
Korea/2013–2017	*R. raoultii*	*Ixodes nipponensis*	Korean water deer	Molecularly and phylogenetically	16S rRNA and *gltA*	[82]
		*Ha. longicornis*				
Romania/2013–2014	*R. raoultii*	*De. marginatus*	Humans	Molecularly (sequencing)	23S rRNA	[83]
Romania/2013	*R. raoultii*	*De. reticulatus*	Dogs	Molecularly (sequencing)	*ompB*	[84]
Poland/2013	*R. raoultii*	*Ix. ricinus*	Vegetation	Molecularly and phylogenetically	*ompA, 16S rRNA,*	[85]
		*De. reticulatus*				
Poland/2013–2014	*R. raoultii*	*De. reticulatus*	Dogs and cats	Molecularly	*gltA*	[86]
Ukraine/2013–2014	*R. raoultii*	*De. reticulatus*	Vegetation	Molecularly	*sca4,*	[87]
France/2014–2021	*R. raoultii*	*De. marginatus*	Humans	Molecularly	*gltA*	[88]
Greece/2014	*R. raoultii*	*De. marginatus*	Goats	Molecularly and phylogenetically	*Atp, gltA, DnaA* and DnaK	[89]
China–Russian border/2014	*R. raoultii*	*Ixodes persulcatus*	Vegetation	Molecularly (sequencing)	*gltA*, *ompA*	[17]
Algeria/2014	*R. raoultii*	*Ix. ricinus*	Cattle	Molecularly		[90]
Netherlands/2014	*R. raoultii*	*De. reticulatus*	Vegetation	Molecularly	*gltA*	[91]
Mongolia/2015–2016	*R. raoultii*	Blood	Human	Molecularly and phylogenetically	16S rRNA, *gltA*, and *ompA*	[92]
		*Hyalomma asiaticum*	Sheep, cattle, camels, dogs			
		*De. nuttalli*				
Serbia	*R. raoultii*	*De. reticulatus*	Dogs	Molecularly	*ompA*	[93]
Austria/2015	*R. raoultii*	*De. reticulatus*	Vegetation	Molecularly	*ompA, gltA*	[94]
China/2015–2016	*R. raoultii*	Serum and blood	Human	Serologically and molecularly	*rrs*, *gltA*, *ompA*, *ompB*, and *sca4*	[29]
Kazakhstan/2015	*R. raoultii*	*De. marginatus*	Vegetation	Molecularly and phylogenetically	*ompB*, *ompA*, 23S–5S	[95]
		*De. reticulatus*				
		*Hy. asiaticum*				
Russia	*R. raoultii*	*De. silvarum*	Vegetation	Molecularly and phylogenetically	16S, *ompA*, *ompB*, *sca4*	[96]
		*Haemaphysalis japonica*				
		*Haemaphysalis concinna*				
Russia (Siberia)/2016	*R. raoultii*	Blood	Human	Molecularly and phylogenetically	*gltA*	[33]
		Cerebrospinal fluid				
Poland/2016–2018	*R. raoultii*	*De. reticulatus*	Vegetation	Molecularly and phylogenetically	*gltA*	[97]
Turkey/2016–2019	*R. raoultii*	*Hyalomma aegyptium*	Tortoise	Molecularly (sequencing)	*gltA*	[98]
India	*R. raoultii–*like	*Ha. bispinosa*	Goats	Molecularly and phylogenetically	*htrA*, *gltA*	[99]
Iran/2017–2018	*R. raoultii*	*Hy. marginatum*	Sheep	Molecularly and phylogenetically	*gltA*	[100]
China/2017	*R. raoultii*	*De. marginatus*	Humans	Serologically and molecularly (phylogenetically)	*17—kDa, gltA, sca1, sca4, ompA,* and *ompB*	[32]
		Blood				
Belgium/2017	*R. raoultii*	*De. reticulatus*	Humans	Molecularly	*gltA*	[101]
China–Kazakhstan border/2017	*R. raoultii*	*De. nuttalli*	Long–tailed ground squirrel	Molecularly and phylogenetically	*17–kDa*, *sca1*, *sca4*, *gltA*, *ompA* and *ompB*	[21]
		*De. silvarum*				
Denmark/2017	*R. raoultii*	*De. reticulatus*	Jackal	Molecularly and phylogenetically	*ompA, gltA, ompB*	[102]
Korea/2018	*R. raoultii*	*Ha. longicornis*	Human	Molecularly and phylogenetically	*ompA*	[22]
Pakistan/2018–2019	*R. raoultii–*like	Blood	Dogs	Molecularly and phylogenetically	*gltA*	[30]
Iran/2018–2019	*R. raoultii*	Blood	Dogs	Molecularly and phylogenetically	*gltA*, *ompA*	[30]
Turkey/2018–2020	*R. raoultii*	*Ctenocephalides felis*	Goats	Molecularly and phylogenetically	*gltA*	[25]
Germany/2018–2019	*R. raoultii*	*De. reticulatus*	Vegetation	Molecularly	*ompB*	[103]
China/2018–2019	*R. raoultii*	*De. marginatus*	Red foxes	Molecularly and phylogenetically	*17–kDa*, *gltA*, *ompA*, *sca1*	[19]
		heart, liver, spleen, lung and kidney				
		*Ixodes canisuga*	Marbled polecat			
Italy/2019	*R. raoultii*	*De. marginatus*	Wild boars	Molecularly and phylogenetically	*ompA*	[104]
China/2019	*R. raoultii*	Blood	Human	Molecularly and phylogenetically	*ompA* and *sca1*	[105]
Romania	*R. raoultii*	*De. marginatus*	Dogs	Molecularly (sequencing)	*gltA*, *17–kDa*	[106]
		*Ix. ricinus*				
		*Rhipicephalus rossicus*				
		*Haemaphysalis punctata*	Vegetation			
China/2019–2020	*R. raoultii*	*Ix. persulcatus*	Human	Molecularly and phylogenetically	*gltA* and *ompA*	[107]
		*De. silvarum*				
		*Ha. concinna*				
India/2020	*R. raoultii*	*Haemaphysalis intermedia*	Cows, goats, and dogs	Molecularly and phylogenetically	*16s rRNA, gltA, ompA,* and *ompB*	[108]
Poland/2021–2022	*R. raoultii*	*De. reticulatus*	Humans	Molecularly (sequencing)	*gltA* and *ompB*	[109]
China/2021–2022	*R. raoultii*	*De. silvarum*	Sheep	Molecularly and phylogenetically	*rrs, gltA, ompA,* and *ompB*	[110]
Siberia/2022	*R. raoultii*	*Dermacentor* spp.	Vegetation	Molecularly and phylogenetically	*gltA, ompA, ompB, htrA,* and *16S rRNA*	[111]

**Table 3:** Table showing the number of various inspected hosts in different localities, collected tick species and their life stages, and molecularly analyzed ticks for the detection of *Rickettsia raoultii* through *gltA, ompA*, and *ompB* fragments.

District	Host			Tick Species	Nymph (%)	Female (%)	Male (%)	Total (%)	Subjected for Molecular Analysis (N, F)	Detection of *Rickettsia raoultii*		
	Type	Examined	Infested							*gltA*	*ompA*	*ompB*
Kohat	Camels	20	14	*Hyalomma dromedarii*	7 (41.2)	6 (35.3)	4 (23.5)	17 (3.0)	2,2	─	─	─
				*Hyalomma isaaci*	15 (45.5)	12 (36.4)	6 (18.2)	33 (5.8)	2,2	─	─	─
				*Hyalomma turanicum*	20 (60.6)	9 (27.3)	4 (12.1)	33 (5.8)	2,2	1N, 1F	1N, 1F	1N, 1F
	Sheep	19	12	*Hy. turanicum*	2 (33.3)	2 (33.3)	2 (33.3)	6 (1.1)	2,2	─	─	─
				*Hy. isaaci*	2 (40.0)	2 (40.0)	1 (20.0)	5 (0.9)	2,2	─	─	─
				*Hyalomma anatolicum*	3 (50.0)	2 (33.3)	1 (16.7)	6 (1.1)	2,2	─	─	─
	Goats	16	14	*Hy. isaaci*	3 (42.9)	2 (28.6)	2 (28.6)	7 (1.2)	2,2	─	─	─
				*Rhipicephalus turanicus*	9 (60.0)	4 (26.7)	2 (13.3)	15 (2.7)	2,2	─	─	─
				*Rhipicephalus microplus*	5 (41.7)	4 (33.3)	3 (25.0)	12 (2.1)	2,2	─	─	─
Total		55	40 (72.7%)		66N	43F	25M	134 (23.8%)	18N, 18F	1N, 1F		
D.I Khan	Camels	18	12	*Hy. dromedarii*	8 (44.4)	6 (33.3)	4 (22.2)	18 (3.9)	2,2	─	─	─
	Sheep	16	9	*Hy. anatolicum*	11 (47.8)	8 (34.8)	4 (17.4)	23 (4.1)	2,2	─	─	─
				*Hy. turanicum*	4 (44.4)	3 (33.3)	2 (22.2)	9 (1.6)	2,2	1N, 1F	1N, 1F	1N, 1F
				*Rh. turanicus*	12 (48.0)	9 (36.0)	4 (16.0)	25 (4.4)	2,2	─	─	─
				*Rhipicephalus haemaphysaloides*	7 (46.7)	5 (33.3)	3 (20.0)	15 (2.7)	2,2	─	─	─
	Goats	14	9	*Hy. anatolicum*	7 (53.8)	4 (30.8)	2 (15.4)	13 (2.3)	2,2	─	─	─
				*Rh. turanicus*	14 (58.3)	6 (25.0)	4 (16.7)	24 (4.2)	2,2	─	─	─
				*Rh. microplus*	4 (40.0)	3 (30.0)	3 (30.0)	10 (1.8)	2,2	─	─	─
Total		48	30 (62.5%)		67N	44F	26M	137 (24.3%)	16N, 16F	1N, 1F		
Lower Dir	Camels	21	14	*Hy. dromedarii*	5 (50.0)	3 (30.0)	2 (20.0)	10 (1.8)	2,2	─	─	─
	Sheep	15	8	*Haemaphysalis* *cornupunctata*	8 (57.1)	4 (28.6)	2 (14.3)	14 (2.5)	2,2	1N	1N	1N
				*Haemaphysalis sulcata*	20 (58.8)	6 (17.6)	8 (23.5)	34 (6.0)	2,2	─	─	─
				*Rh. microplus*	5 (45.5)	4 (36.4)	2 (18.2)	11 (1.9)	2,2	─	─	─
	Goats	13	6	*Ha. cornupunctata*	5 (55.6)	2 (22.2)	2 (22.2)	9 (1.6)	2,2	1N	1N	1N
				*Rh. haemaphysaloides*	8 (66.7)	2 (16.7)	2 (16.7)	12 (2.1)	2,2	─	─	─
Total		49	28 (57.1%)		51N	21F	18M	90 (16.0%)	12N, 12F	2N		
Bajaur	Camels	17	11	*Hy. dromedarii*	7 (50.0)	4 (28.6)	3 (21.4)	14 (2.5)	2,2	─	─	─
	Sheep	17	9	*Haemaphysalis bispinosa*	7 (46.7)	5 (33.3)	3 (20.0)	15 (2.7)	2,2	1N	1N	1N
				*Haemaphysalis sulcata*	10 (52.6)	6 (31.6)	3 (15.8)	19 (3.4)	2,2	─	─	─
				*Ha. cornupunctata*	5 (38.5)	5 (38.5)	3 (23.1)	13 (2.3)	2,2	─	─	─
	Goats	16	8	*Ha. bispinosa*	9 (60.0)	4 (26.7)	2 (13.3)	15 (2.7)	2,2	─	─	─
				*Rh. microplus*	5 (41.7)	4 (33.3)	3 (25.0)	12 (2.1)	2,2	─	─	─
				*Ha. cornupunctata*	8 (61.5)	3 (23.1)	2 (15.4)	13 (2.3)	2,2	─	─	─
Total		50	28 (56.0%)		51N	31F	19M	101 (17.9%)	14N, 14F	1N		
Mansehra	Camels	23	13	*Hy. dromedarii*	6 (46.2)	4 (30.8)	2 (15.4)	13 (2.3)	2,2	─	─	─
	Sheep	18	10	*Rh. haemaphysaloides*	5 (50.0)	3 (30.0)	2 (20.0)	10 (1.8)	2,2	─	─	─
				*Ha. bispinosa*	4 (50.0)	2 (25.0)	2 (25.0)	8 (1.4)	2,2	─	─	─
				*Haemaphysalis montgomeryi*	15 (55.6)	9 (33.3)	3 (11.1)	27 (4.8)	2,2	1N	1N	1N
	Goats	18	12	*Ha. sulcata*	8 (47.1)	6 (35.3)	3 (17.7)	17 (3.0)	2,2	─	─	─
				*Rh. microplus*	5 (50.0)	3 (30.0)	2 (20.0)	10 (1.8)	2,2	─	─	─
				*Ha. montgomeryi*	9 (52.9)	5 (29.4)	3 (17.7)	17 (3.0)	2,2	─	─	─
Total		59	35 (59.3%)		52N	32F	18M	102 (18.1%)	14N, 14F	1N		
Overall total		261	161 (61.7%)		287N (50.9%)	171F (30.3%)	106M(18.8%)	564	74N (25.8%), 74F (43.3%)	6N (2.1%), 2F (3.5%)		

## Data Availability

All data generated or analyzed during this study were included in this article. Further inquiries can be directed to the corresponding authors.
